# An anti-inflammatory diet intervention for knee osteoarthritis: a feasibility study

**DOI:** 10.1186/s12891-022-05003-7

**Published:** 2022-01-13

**Authors:** Indiana Cooper, Peter Brukner, Brooke L. Devlin, Anjana J. Reddy, Melanie Fulton, Joanne L. Kemp, Adam G. Culvenor

**Affiliations:** 1 Melbourne Medical School, University of Melbourne, Parkville, Australia; 2 La Trobe Sport and Exercise Medicine Research Centre, School of Allied Health, Human Services and Sport, La Trobe University, Bundoora, Victoria 3086 Australia; 3 Department of Dietetics, Nutrition and Sport, La Trobe University, Melbourne, Australia

**Keywords:** Osteoarthritis, Knee Joint, Anti-inflammatory diet, Mediterranean diet, Low-carbohydrate diet, Telehealth

## Abstract

**Background:**

Knee osteoarthritis has an inflammatory component that is linked to pain and joint pathology, yet common non-surgical and non-pharmacological interventions (e.g., exercise, calorie restricting diets) do not typically target inflammation. We aimed to evaluate the feasibility of a telehealth delivered anti-inflammatory diet intervention for knee osteoarthritis.

**Methods:**

This 9-week single-arm feasibility study recruited participants aged 40–85 years with symptomatic knee osteoarthritis (inclusion criteria: average pain ≥4/10 or maximal pain ≥5/10 during past week). All participants received a telehealth-delivered anti-inflammatory dietary education intervention involving 1:1 consultations at baseline, 3- and 6-week follow-up. The diet emphasised nutrient-dense wholefoods and minimally processed anti-inflammatory foods and discouraged processed foods considered to be pro-inflammatory. The primary outcome of feasibility was assessed via: i) eligibility, recruitment and retention rates; ii) self-reported dietary adherence; iii) adverse events; and iv) treatment satisfaction. Post-intervention interviews evaluated the acceptability of the dietary intervention delivered via telehealth. Secondary outcomes included changes in self-reported body mass, Knee injury and Osteoarthritis Outcome Score (KOOS), health-related quality of life (EuroQoL-5D), analgesic use and global rating of change. Worthwhile effects were determined by the minimal detectable change (MDC) for all five KOOS-subscales (pain, symptoms, activities of daily living, sport/recreation, quality of life) being contained within the 95% confidence interval.

**Results:**

Forty-eight of seventy-three (66%) individuals screened were eligible and 28 enrolled over 2 months (82% female, mean age 66 ± 8 years, body mass index 30.7 ± 4.8 kg.m^−2^). Six participants withdrew prior to final follow-up (21% drop-out). Of those with final follow-up data, attendance at scheduled telehealth consultations was 99%. Self-reported adherence to diet during the 9-week intervention period: everyday = 27%, most of time = 68% and some of time = 5%. Two minor adverse events were reported. Change scores contained the MDC within the 95% confidence interval for all five KOOS subscales. Suggestions to improve study design and limit drop-out included an initial face-to-face consultation and more comprehensive habitual dietary intake data collection.

**Conclusion:**

This study supports the feasibility of a full-scale randomised controlled trial to determine the efficacy of a primarily telehealth-delivered anti-inflammatory dietary education intervention in adults with symptomatic knee osteoarthritis.

**Trial registration:**

ACTRN12620000229976 prospectively on 25/2/2020.

**Supplementary Information:**

The online version contains supplementary material available at 10.1186/s12891-022-05003-7.

## Background

Knee osteoarthritis (OA) is the leading cause of global disability in the elderly and carries a tremendous health and economic burden [1]. In Australia alone, OA-related healthcare costs exceed $2.1 billion annually [2, 3]. With no cure or regulatory approved disease-modifying drugs, treatment for OA is largely symptomatic [4]. Surgical joint replacement is an effective procedure in the right candidate but is limited to those with end-stage joint disease, and up to 20% of patients have no clinically meaningful improvement [5, 6].

Clinical guidelines recommend exercise-therapy and weight-loss as first-line treatments for knee OA that target typical physical impairments (e.g., muscle weakness, excessive joint loads) [7]. Exercise-therapy is supported by more than 50 randomised controlled trials (RCTs), yet its effect on pain and quality of life is only moderate [7, 8]. Weight loss of at least 5–10% body weight has been shown to improve OA-related symptoms and function [8–11]. However, typical dietary interventions are caloric restrictive, which can hinder compliance and long-term sustainability [12]. A recent meta-analysis highlighted that, within two years, more than half of weight lost was regained, and by 5 years, this figure jumps to over 80% [13].

Anti-inflammatory diets provide an alternative to caloric restrictive approaches by targeting local and systematic inflammation, both contributors to OA disease onset, progression and symptom burden [14–16]. In recent years, diets high in anti-inflammatory properties have garnered significant interest in the prevention and management of chronic diseases [17]. Typically, these diets are high in unrefined and minimally processed foods, dense in nutrients including fibre, monounsaturated and polyunsaturated fatty acids (MUFAs/PUFAs) and have been shown to significantly reduce inflammation independent of weight loss [17–19]. Consumption of foods rich in polyphenols such as fruits, vegetables, herbs, spices, and olive oil can decrease inflammation via antioxidant and anti-inflammatory properties which neutralise free radicals and other reactive oxygen species [20]. Alternatively, ultra-processed foods with a high glycaemic load, such as refined carbohydrates (breads, grains, starchy vegetables, junk foods), can increase the production of free radicals and proinflammatory cytokines, leading to a pro-inflammatory milieu [21]. Omega-3 fatty acids are also a key nutrient within an anti-inflammatory diet, with nuts, seeds and fish being a rich source for omega-3 fatty acids [22]. Diets rich in omega-3 fatty acids are crucial for achieving a more desirable omega-6 to omega-3 ratio in a healthy diet. In contrast omega-6 fatty acids, can be converted into arachidonic acid, contributing to precursors for proinflammatory eicosanoids [21]. An elevated omega-6:omega-3 ratio mediates vascular damage and reduces anti-inflammatory processes, likely exacerbating oxidative stress, which increases the risk and severity of chronic disease, including OA [23–25].

Small studies (i.e., <50 participants) that have included anti-inflammatory diets as an intervention for knee OA provide preliminary indications that they are feasible and effective at reducing symptoms and inflammation associated with knee OA over 12–16 weeks [25, 26]. However, these studies relied upon regular and intensive face-to-face consultations. With the recent global pandemic (COVID-19), there have been calls for evaluations of the effectiveness of remotely delivered healthcare (i.e., telehealth), including dietary interventions [27, 28]. It is important to establish the feasibility of a telehealth-delivered anti-inflammatory dietary intervention prior to undertaking a full-scale RCT.

The primary aim of this study was to determine the feasibility of a full-scale RCT to estimate the effectiveness of an anti-inflammatory dietary intervention delivered via telehealth. Our secondary aim was to determine if a worthwhile effect was observed for improvements in self-reported knee symptoms, function, and quality of life.

## Methods

### Study design

This single-arm feasibility trial was conducted at La Trobe University, Melbourne, Australia. The trial was prospectively registered with the Australian New Zealand Clinical Trial Registry (ACTRN12620000229976) and reporting adheres to the Consolidated Standards of Reporting Trials (CONSORT) statement for pilot and feasibility studies [29]. Ethical approval was gained from La Trobe University Human Ethics Committee (HEC19525) and all participants provided written informed consent prior to enrolment. All methods were carried out in accordance with relevant guideline and regulations. All patient-reported outcomes were completed via an online Research Electronic Data Capture (REDCap) platform.

While we initially planned to randomise participants into one of two groups (anti-inflammatory diet vs no-intervention control), and collect biochemical (i.e., serum inflammatory markers), body composition (i.e., Dual energy X-ray absorptiometry (DEXA) scan) and physical performance outcomes (i.e., 30 m walk test, 20 s chair stand test), we modified our study protocol prior to the first enrolled participant due to government and university COVID-19 restrictions so that all outcomes were self-reported and completed remotely.

### Participant recruitment and eligibility

We initially aimed to enrol 60 participants (*n* = 30 anti-inflammatory diet, *n* = 30 control) based on previous feasibility trials evaluating health-professional guided interventions for musculoskeletal conditions [30–32] which was deemed sufficient to assess feasibility parameters. Due to COVID-19, our study was adapted to a single-arm feasibility study, in which we aimed to enrol 30 participants into the anti-inflammatory diet intervention. Between February and April 2020, study information was distributed via an online newsletter to individuals on a registry who had completed an exercise-therapy program for OA throughout Australia (i.e., GLA:D Australia) [33]. Individuals contacted the research team to undergo eligibility screening via phone.

Inclusion criteria were: (i) aged 40 to 85 years; (ii) average knee pain of ≥4/10 on a numeric rating scale or maximum intensity of ≥5/10 in past 7 days; (iii) ability to understand written and spoken English; (iv) willing to follow a 9-week anti-inflammatory diet. Exclusion criteria were: (i) knee pain not primarily due to OA (e.g., fibromyalgia, tumour, referred pain); (ii) already participating in a specific diet (e.g., low carbohydrate high-fat, Paleo, Mediterranean); (iii) unstable weight (>5 kg weight change in past 3 months).

### Anti-inflammatory diet intervention

The anti-inflammatory diet intervention was administered by Accredited Practising Dietitians (APD) or by researchers who were specifically trained by accredited dietitians. Standardised case report forms were used during each telehealth consultation to maximise standardisation of the dietary intervention.

The dietary intervention was delivered over 9-weeks, with telehealth consultations via Zoom platform (Zoom Video Communications, Inc., San Jose, Ca, North America, Version: 5.0.1) for all baseline consultations, with the option for either Zoom or telephone consultations (based on participant preference) at 3- and 6-weeks (Table 1). Baseline consultations were conducted over 45–90 min depending on participant understanding of anti-inflammatory diets and completion of food diaries. Follow-up consultations were conducted over 10–15 min. The baseline consultation consisted of education regarding the intervention and answering participant questions. Participants were encouraged to follow a diet containing minimal processed foods and higher amounts of “good” fats and wholefoods. The wholefoods encouraged in moderate amounts were: lean meats, eggs and dairy; and those encouraged in higher amounts were: fish, fruit, vegetables, nuts and seeds. “Good fats” included monounsaturated fats with a favourable omega-6:omega-3 ratio such as fish, seeds and olive oil. Participants were requested to limit highly processed and refined foods such as refined carbohydrates (pasta, bread, rice), confectionary and processed meats. Participants were encouraged to consume a normocaloric diet and to eat to satiety. Information provided via telehealth was supplemented by a study booklet that was mailed to participants detailing all dietary advice and examples of food to consume and avoid (Additional file 1). Participants were instructed not to partake in any other knee OA intervention during the 9-week study period, other than stable medication doses.

**Table 1:** Overview of the anti-inflammatory diet intervention^a^

Name	Anti-inflammatory diet intervention
What	Education and discussion 1-to-1 supplemented with a study booklet of examples of foods to consume and recipes
Who provides	Accredited Practising Dietitian or researchers (trained by dietitian to deliver the intervention).
How	1-to-1 telehealth sessions via Zoom or telephone consult (when video teleconferencing was not available for follow-up appointments). All baseline appointments were delivered by telehealth videoconferencing.
Where	Remotely conducted telehealth sessions by researchers in Melbourne to participants throughout Australia.
When & how much	Telehealth 1-to-1 sessions: baseline, 3- and 6-week follow-up. Baseline: 45–90 min. Follow-ups: 10–15 min.
Tailoring	• Dietary education provided including list of acceptable food groups and possible adverse outcomes. • Standardised meal plan and shopping list provided, however, encouraged acceptable modifications to suit individual lifestyle and palate. • Individualised feedback and education provided at each follow-up after assessment of most recent 3-day food diary. • Individualised education provided at each follow-up for participants who had specific questions regarding their food intake and acceptable foods.
How well	Attendance at telehealth sessions recorded by the intervention dietitian or trained researcher. Self-reported dietary adherence recorded on a 5-point Likert scale (ranging from never adherent to adherent every day).

^a^Described according to the Template for Intervention Description and Replication [34]

To guide education content, data from a validated multiple pass 24-h food recall (completed at baseline) [35], and a validated 3-day food diary (completed at 3-, 6- and 9-weeks), were used to provide feedback and discuss participant-specific strategies to optimise adherence. The multiple pass 24-h food recall and 3-day food diaries were analysed using FoodWorks10® (Xyris Software, Brisbane, QLD, Australia) incorporating the AUSNUT 2013, AusBrands 2015 and AusFoods 2015 databases. Participants were given the option of either paper-based recording for the 3-day food diaries, or were taught to use a smartphone-based application, *Easy Diet Diary.* Easy Diet Diary is a commercial food diary and calorie counter that is developed and owned by Xyris Software, Brisbane, QLD, Australia). Macronutrient, micronutrient and food group analysis was exported from FoodWorks10®.

### Outcomes

#### Baseline characteristics

Participant characteristics (e.g., age, sex, symptom duration, education, employment, income, physical activity, current diet) were collected at baseline. Symptom duration was answered by the question: “In the most affected knee, what is the duration of your knee pain?”. Symptom duration was then split into four categories from 0–6 months to >3 years. Baseline diet was assessed with the multiple pass 24-h food recall [35]. The timepoints of all outcome measure collection are presented in Table 2.

**Table 2:** Overview of data collection

Variable	Baseline	Week 3	Week 6	Week 9
Ethnicity	X			
Highest education level	X			
Employment status	X			
Civil status	X			
Living situation	X			
Comorbidities	X			
Knee symptom history	X			
24-h recall food diary	X			
Current knee pain	X	X	X	X
Height and weight	X	X	X	X
EuroQoL-5D	X	X	X	X
Knee injury and Osteoarthritis Outcome Score	X	X	X	X
Analgesic medication use	X	X	X	X
3-day food diary		X	X	X
Adverse events	X	X	X	X

#### Primary outcome: Feasibility

Feasibility was assessed according to previously published recommendations [36] and included the following parameters: (i) eligibility rate; (ii) recruitment rate; (iii) retention rate; iv) dietary adherence; v) 3-day food diary completion; vi) attendance at telehealth consults and vii) occurrence of adverse events. Proceeding to a full-scale RCT was considered feasible where parameters were comparable to previously published recommendations, or reasonable amendments could be applied to achieve these results in future trials.

Dietary adherence was assessed via a 5-point Likert scale asking how often the dietary intervention was followed (never to every day), which was supplemented by completion of the 3-day food diary at each follow-up to assess specific dietary intake. Adverse events were defined as those resulting in new limitations to normal daily activities, recreational- or work-related activities, or symptoms requiring medical care.

We also assessed acceptability, accessibility, adherence, and treatment satisfaction during semi-structured interviews following the intervention. Interviews were conducted by a single researcher and recorded via Zoom (Zoom Video Communications, Inc., San Jose, CA, North America, Version: 5.0.1). Responses to 20 open-ended questions (Additional File 2) covering themes such as: (i) acceptability of dietary intervention; (ii) accessibility of telehealth consultations; (iii) adherence to dietary intervention; (iv) treatment satisfaction; and (iv) comparison to exercise-based interventions. The interviews were transcribed verbatim and explored using thematic analysis [37]. Participant responses were then coded in an inductive manner. Post-analysis, the themes were verified between two investigators (IC, MF).

#### Secondary outcome: Knee symptoms

Knee symptoms were assessed using the Knee injury and Osteoarthritis Outcome Score (KOOS) [38]. The KOOS is a 42-item patient-reported outcome measure consisting of five subscales: Pain, Symptoms, Activities of Daily Living (ADL), Function in Sport and Recreation (Sport/Rec), and Quality of Life (QoL). Participants rate each item on five graded adjectival response options, then mean scores for each subscale are calculated and converted to be expressed as a score ranging from 0 to 100, with 100 representing no problems. KOOS_4_, the mean score of four of the five subscales (all except Sport/Rec) will also be assessed as this has been used as a primary outcome in trials of knee OA [39]. KOOS is a valid, reliable, and responsive measure during short-term and long-term follow-up for knee OA [40]. We also assessed self-reported knee pain during the previous 7 days using a 100 mm visual analogue scale (VAS) for both average knee pain and most severe knee pain.

#### Secondary outcome: health-related quality of life

Health-related quality of life was assessed with the EuroQol-5D (EQ-5D), which comprises five health domains (mobility, self-care, usual activities, pain and anxiety/depression) as well as a VAS for overall health status from 0 (worst) to 100 (best) [41]. Responses for the five health domains were combined using established formula to provide an overall health-related quality of life index value [42].

#### Secondary outcome: analgesic medication

Change in analgesic medication use from baseline to 9-week follow-up was assessed with a 7-point Likert scale (much less to much more).

#### Secondary outcome: body mass

Weight (kg) and height (cm) were self-reported by participants following advice regarding how to accurately assess these, ensuring consistent weighing times and conditions (e.g., before first meal) and BMI (kg.cm^−2^) was calculated.

### Data analysis

Participants who completed baseline and 9-week follow-up assessments (primary study endpoint) were included in the analysis, as per CONSORT recommendations [29]. Feasibility outcomes were reported descriptively. Within-group change in secondary continuous outcomes were reported as mean (95% confidence interval (CI)) change and evaluated with paired t-tests. For non-normally distributed data, a Wilcoxon Signed Rank test assessed pre-post-intervention differences. Treatment effects were potentially worthwhile if previously estimated minimal detectable change (MDC) scores for each measure were contained within the 95% CI. We used the macronutrient, micronutrient, and food group analysis data from FoodWorks10® to calculate changes in dietary intake over time. Normally distributed intake data (confirmed with Kolmogorov-Smirnov test) are reported as mean ± standard deviation (SD) and differences evaluated with paired t-tests. Non-normally distributed intake data are reported as median (interquartile range (IQR)) and change overtime evaluated with Wilcoxon Signed Rank test. Statistical analyses were conducted in Stata (StataCorp, V.16.0) with α = 0.05.

## Results

### Feasibility

**Fig. 1:**
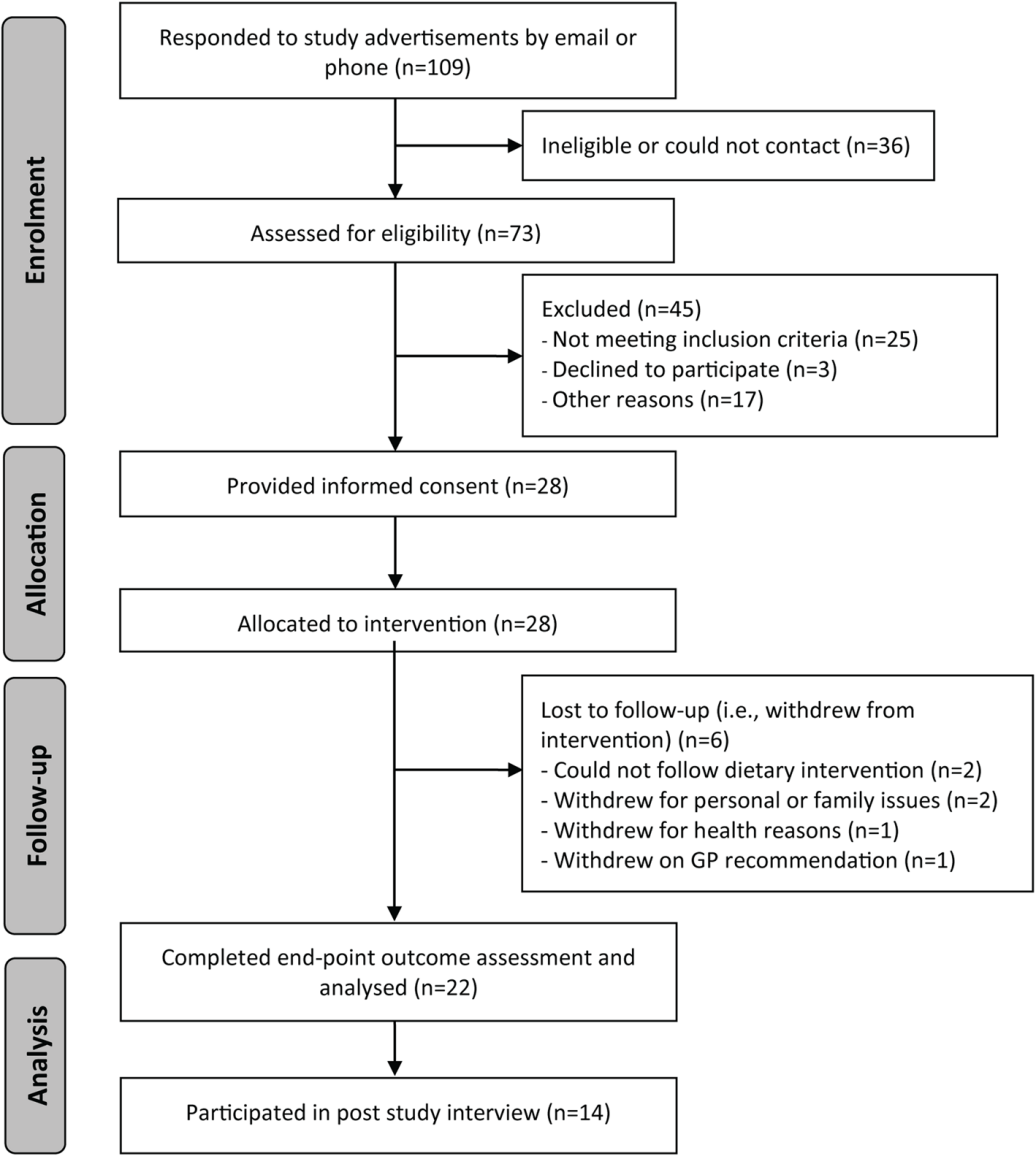
Flow of participants through the study

During March and April 2020, 109 individuals responded to the study invitation. Seventy-three (67%) individuals were screened, with 48 (64%) of those meeting eligibility criteria. Of the eligible participants, 28 (58%) were enrolled. Twenty-two participants completed the entire 9-week dietary intervention and final follow-up assessments, with six withdrawals (drop-out rate 21%: *n* = 2 could not follow intervention, *n* = 2 due to personal reasons, *n* = 1 due to health reasons and *n* = 1 based on GP recommendation) (Fig. 1). The results of each aspect of feasibility are summarised in Table 3. There were two adverse events reported. One participant developed constipation, which resolved after further dietary advice given at the 3-week follow-up appointment, and a second participant had an injury to the knee following a fall, which the participant reported as unrelated to the diet.

**Table 3:** Feasibility outcomes

	Anti-inflammatory intervention	Recommendations for full-scale clinical trial
**Recruitment and retention**		
Recruitment rate	14 participants per month	Could be increased utilising physiotherapy and orthopaedic clinics.
Eligibility rate	48 of 73 (66%) screened participants eligible	
Enrolment rate	28 of 48 (58%) of eligible participants enrolled	
Drop-out rate	6 (21%)	Strategies required to improve drop-out rate may include better education of intervention and follow-up requirements prior to enrolment, having a patient ambassador or using an interactive mobile app to optimise engagement.
**Adherence and attendance**		
Dietary adherence	96% reported adherence on the Likert scale of ≥4/5 at final follow-up	Increased meal plans/recipes. Utilisation of interactive food recording tools.
Telehealth attendance	99% consult attendance	
Food Diary completion	100% completion of diaries	
**Adverse events**		
Injury or illness	*N* = 2 (1 constipation, 1 increased knee pain following fall)	Could incorporate more overt preventive strategies for constipation.
**Acceptability of outcomes**		
Treatment satisfaction	Participants reported appointments were appropriate regarding: availability, frequency and duration. Participants were satisfied with the diet intervention with most (86%) of interviewed participants stating they would continue the diet.	Consider initial baseline face-to-face consultation with tele-health follow-up.
Time to collect data	Baseline appointments completed in <90 min. Follow-up appointments completed in 10–15 min.	
Completeness of patient-reported outcomes	Of the 22 participants attending the 9-week follow-up, with a total of 88 data collection events (4 each): - Missing data n = 1 (1%) - Incomplete data n = 6 (7%)	Data checking mechanisms to reduce incomplete data.
Adherence monitoring	15 participants used *Easy Diet Diary*, 7 used paper food records. Participants reported food diary was useful for motivation and accountability. Participants reported that the macronutrient tracker on the *Easy Diet Diary* was helpful in providing real-time analysis of foods consumed, which aided food choices.	

Of the 22 participants with 9-week follow-up data, 6 (27%) reported being adherent every day, 15 (68%) reported being adherent most of the time and 1 (4.5%) reported being adherent sometimes. Post-study interviews (*n* = 14) revealed that participants were generally satisfied with the dietary intervention. Eighty-six percent reported that they were likely to continue following the diet after study completion with the remaining 14% stating they would likely continue with a modified version of the diet. Example quotes from the themes of accessibility, acceptability and adherence are provided in Table 4 and Additional file 3.

**Table 4:** Supporting quotes from post-intervention interviews exploring the intervention accessibility, acceptability and adherence

**Theme 1: Accessibility of telehealth appointments**	
* Quote 1: I suppose the fact that you didn’t have to travel to an appointment and find extra time to put into it was good.*	
* Q2: It was good to have some flexibility if a time* [for a telehealth appointment] *didn’t suit.*	
* Q3: If possible in the future you have the* [initial] *face-to-face, do all that* [baseline testing] *and the rest of it would be just zoom.*	
* Q4: Yep. That was good* [flexible appointment scheduling with telehealth], *because I’m a shift worker, so it was good to have some flexibility if a time didn’t suit.*	
* Q5: It* [follow up appointments] *didn’t need to be face-to-face and lucky for that because we didn’t have any face-to-face opportunities* [due to COVID-19 restrictions]. *But still, like with using Zoom and all that, it’s nearly like sitting in the same room anyhow.*	
* Q6: Once I got my head around the Zoom, sort of sorted that out, in my brain, it was fine. I’ve got no issue with it* [using telehealth instead of face-to-face].	
**Theme 2: Acceptability of the dietary intervention**	
* Q7: Actually, throughout the diet I found that, when I adhered to it [anti-inflammatory diet], I was far less likely to eat between meals, which was good.*	
* Q8: Yes. I’m going to continue the diet because I felt better.*	
* Q9: Yeah, I will* [continue the diet]. *Because, clearly my pain levels have diminished and, yeah, I’m not a big carbohydrate eater anyway, so if I don’t have those foods, it won’t bother me.*	
* Q10: Yeah. Generally, I really enjoyed it* [the dietary intervention]	
* Q11: ..After two or three weeks, it was quite enjoyable and the recipes that you provided and everything were really nice. So, that makes it easier, to have something tasty*	
* Q12: … I would recommend it to anybody who has got the problems* [osteoarthritis*] that I have got.*	
**Theme 3: Adherence to the dietary intervention**	
* Q13: I think everyday I adhered to it.*	
* Q14: Perfect … I was perfect* [adhering to diet]. *I didn’t do anything naughty at all.*	
* Q15: I suppose, when I was having positive results out of it, when my knee was not hurting as much, when I could actually see that the swelling on my knee.. was going down, plus seeing the weight loss and everything. So, it wasn’t hard to adhere to it.*	
* Q16: I was really pleased we were in lockdown, I must admit. I think that would have been much more difficult to maintain* [adherence to diet] *going in and out of work everyday.*	
**Theme 4: Comparison to exercise-intervention**	
* Q17: Oh, the diet was easier to follow* [compared to exercise intervention]. *The diet was much easier to follow.*	
* Q18: It was easier to follow the eating plan. Because I did GLA:D* [exercise intervention] *post a total knee replacement. So, that was hard for pain.*	
* Q19: Oh, it* [diet] *was much easier because I find the exercises very tiring and hard.*	
* Q20: I didn’t really find the diet that hard to stick to. So, I would probably sort of say equal.*	

### Participant characteristics

The 28 enrolled participants were mostly women (82%), mean age 66 ± 8 years, who were overweight (body mass index 30.6 ± 4.6) (Table 5). All participants were Caucasian and most had pre-existing comorbidities (85%) and knee pain persisting for more than one year (67%).

**Table 5:** Overview of baseline participant characteristics

Variable	Total (***n*** = 27)^#^
Age, mean ± standard deviation years	66 ± 8
Female sex, n (%)	22 (82)
Height, mean ± standard deviation cm^a^	166.6 ± 8.2
Weight, mean ± standard deviation kg^a^	84.4 ± 16.0
Body mass index, mean ± standard deviation kg.m^-2a^	30.6 ± 4.6
Caucasian ethnicity, n (%)	27 (100)
Highest education level, n (%)	
Up to secondary school	1 (4)
Completed secondary school	8 (30)
Apprenticeship	3 (11)
Bachelor’s degree	10 (37)
Post-graduate degree	5 (19)
Employment, n (%)	
Full-time	3 (11)
Part-time	5 (19)
Casual	1 (4)
Retired	18 (67)
Living arrangement, n (%)	
Alone	5 (19)
With spouse	18 (67)
With family	4 (15)
Household income $AUD, n (%)	
< 50,000	13 (48)
> 50,000	9 (33)
Undisclosed	5 (19)
Co-morbidities, n (%)	
Diabetes	1 (4)
Hypertension	8 (30)
Dyslipidaemia	5 (19)
Pulmonary disease	1 (4)
Cancer	3 (11)
Other^c^	5 (19)
Knee affected by osteoarthritis, n (%)	
Left	14 (52)
Right	11 (41)
Both	2 (7)
Average knee pain (0–10), mean ± standard deviation^b^	4.8 ± 1.7
Maximal knee pain (0–10), mean ± standard deviation	7.1 ± 1.8
Duration of knee pain, n (%)	
0–6 months	5 (19)
6–12 months	4 (15)
1–3 years	8 (30)
> 3 years	10 (37)
Past knee injury, n (%)	14 (52)
Family history of osteoarthritis, n (%)	18 (67)
Knee	8 (30)
Other joint^d^	10 (37)
Analgesic use, n (%)^b^	22 (82)
Paracetamol	16 (59)
Oral NSAID	6 (22)
Topical NSAID	3 (11)
Glucosamine	3 (11)
Corticosteroid	3 (11)
Opioid	1 (4)
Codeine	2 (7)
Methotrexate	1 (4)
Anti-depressant (chronic pain)	1 (4)

*NSAID* Non-steroidal anti-inflammatory drug

# One participant who withdrew from the study did not consent for their data to be included in the paper

^a^ *n* = 3 missing baseline anthropometry due to no equipment

^b^*n* = 1 data incomplete: Participant did not complete knee pain or analgesic use

^c^ Other medical conditions include coeliac disease, osteoporosis, vascular disease, fibromyalgia

^d^Other osteoarthritis includes: hip, hand, shoulder and back

### Dietary Intake

**Fig. 2:**
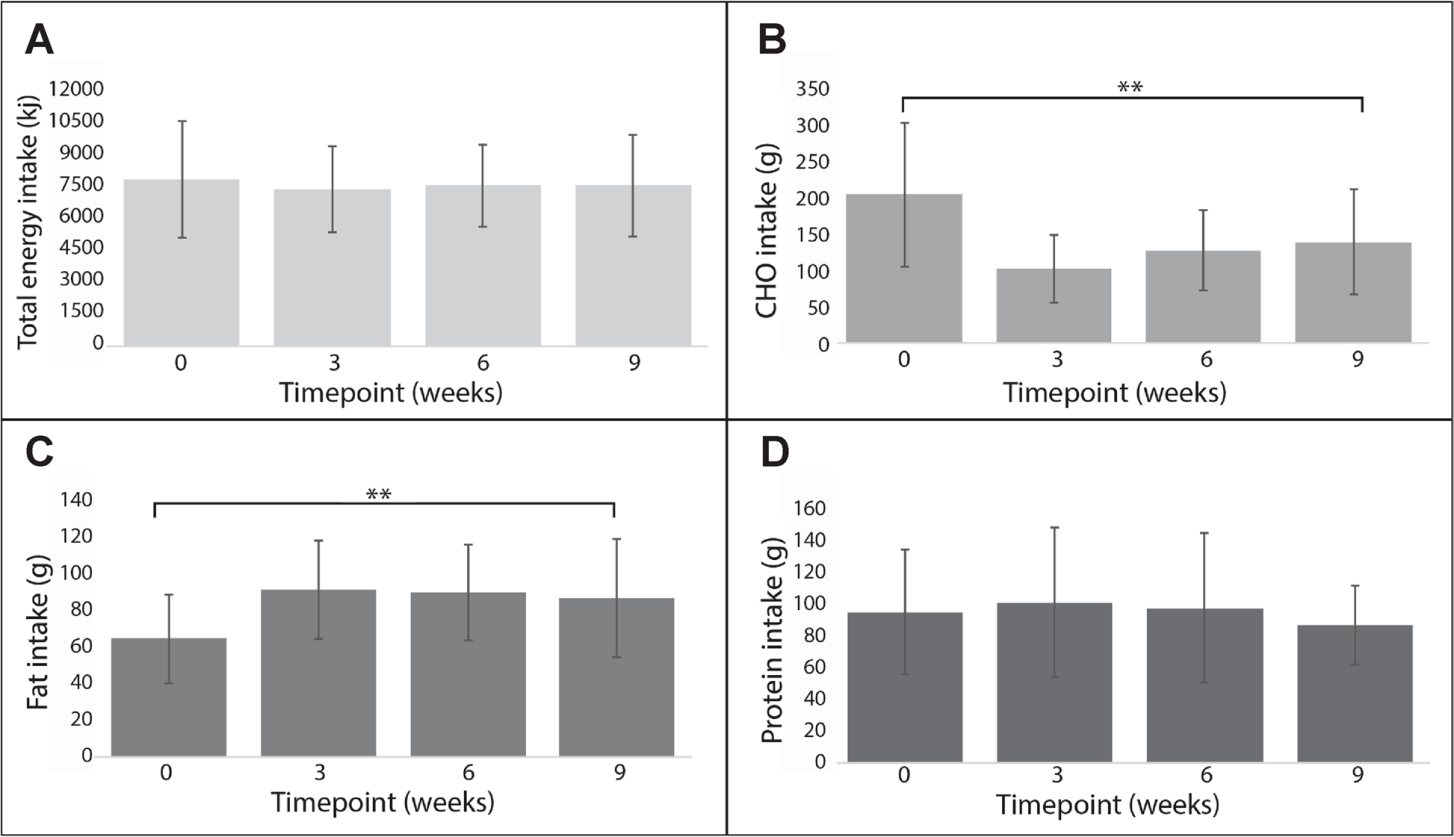
Mean ± standard deviation daily intake in those who completed all follow-ups (*n* = 22). A) Total energy; B) Carbohydrate; C) Fat; D) Protein. ** Indicates significant absolute change in variable from baseline (week 0) to week 9

While overall energy and protein intake remained unchanged between baseline and week 9 (mean change −69.0 kcal [95% CI −308.8 to 170.7] and − 8.1 g [−20.9 to 4.8], respectively), significant reductions were observed in total carbohydrate (−64.8 g [−104.9 to −24.7] (Fig. 2) and carbohydrate as a percent of total energy consumed (−13.3% [−18.2 to −8.4]) (Additional file 4). Total fat intake increased over the 9-week period (22.5 g [7.7 to 37.3]) (Fig. 2) while saturated fat as a percent of total fat intake decreased (−5.7%, [−11.0 to −0.5]), whereas MUFAs and PUFAs percentage intake increased (4.3% [1.3 to 7.4]) and (1.4% [−1.7 to 4.5]), respectively (Additional file 4).

### Patient-reported outcomes

**Fig. 3:**
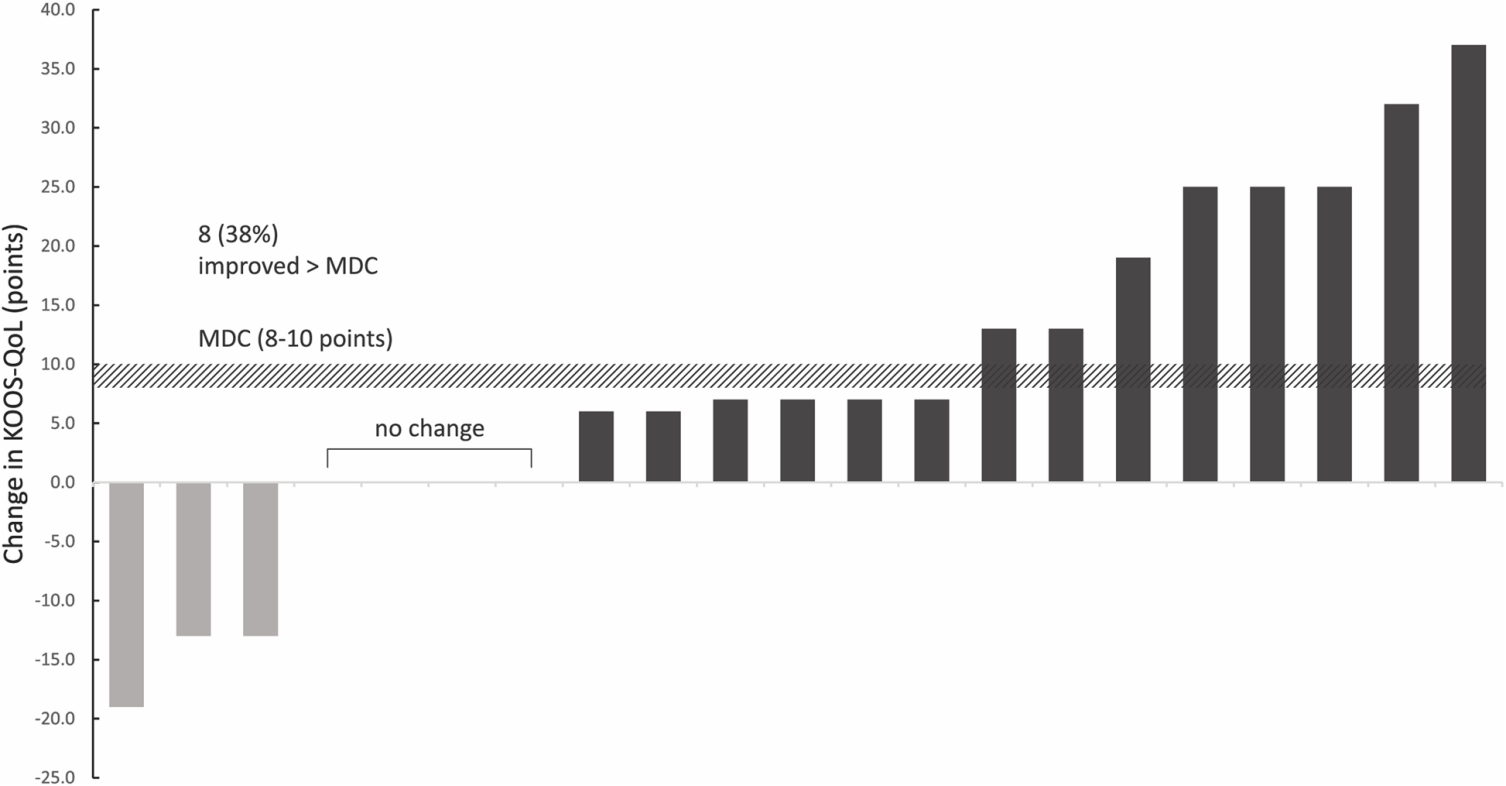
Baseline to week 9 change scores per participant for the KOOS-QoL subscale. Baseline and follow-up KOOS scores range from 0 (extreme problems) to 100 (no problems). Positive change scores indicate an improvement in quality of life. KOOS, Knee injury and Osteoarthritis Outcome Score; MDC, minimal detectable change; QoL, quality of life

The desired treatment effect for all KOOS subscales and KOOS_4_ (improvement >8–10 points) was contained within the 95% CI (Table 6). The individual treatment responses for KOOS-QoL and proportion with improvements greater than the MDC appear in Fig. 3 (other subscales in Additional file 5). Health-related QoL also improved (EQ-5D health utility index mean 6.3, 95% CI −0.44 to 12.7). On average, participants recorded a mean loss of body mass (−3.0 kg, 95% CI −3.6 to −2.3) and decreased BMI (−1.0 kg/m^2^, 95% CI −1.25 to −0.8). Seven participants (32%) reported using much less analgesic medication, 3 (14%) less, 10 (46%) the same amount, and 2 (9%) more (one of whom had an acute flare of pain due to a fall).

**Table 6:** Patient-reported and anthropometric data in participants who completed all follow-up (*n* = 22)

Outcome	Baseline	Week 3	Week 6	Week 9	Change^a^	95% CI of change	MDC
KOOS-Pain	61.8 ± 12.0^b^	63.1 ± 11.4	68.8 ± 12.2^c^	68.4 ± 12.3	6.6 ± 12.6	[0.9 to 12.4]	8–10
KOOS-Symptoms	56.9 ± 14.3^b^	57.3 ± 15.4	58.6 ± 16.7^c^	62.5 ± 17.7	5.6 ± 15.1	[−1.3 to 12.5]	8–10
KOOS-ADL	68.2 ± 15.9^b^	72.2 ± 13.2^b^	75.8 ± 14.6^c^	78.3 ± 14.4	10.1 ± 14.3	[3.6 to 16.6]	8–10
KOOS-Sport/Rec	35.7 ± 27.3^b^	47.4 ± 27.9^b^	46.8 ± 25.2^c^	46.9 ± 29.6	11.2 ± 19.7	[2.2 to 20.2]	8–10
KOOS-QoL	42.0 ± 16.4^b^	46.3 ± 14.6^b^	50.9 ± 14.5^c^	50.8 ± 13.7	8.8 ± 14.7	[2.1 to 15.5]	8–10
KOOS_4_	57.2 ± 10.9^b^	59.95 ± 10.7^b^	63.5 ± 10.8^c^	65.0 ± 12.4	7.8 ± 11.5	[2.5 to 13.0]	8–10
EQ 5 D Utility	0.70 ± 0.16^b^	0.74 ± 0.1	0.74 ± 0.11^c^	0.75 ± 0.12	0.04 ± 0.16	[−0.03 to 0.11]	N/A
EQ 5 D VAS	75.2 ± 15.1^b^	77.5 ± 15.1	78.9 ± 15.4^c^	81.6 ± 14.6	6.3 ± 16.0	[−0.9 to 13.6]	N/A
Average Pain (0-100 mm)	52.6 ± 22.7^b^	52.1 ± 21.3^b^	41.6 ± 23.0^d^	43.6 ± 27.5^c^	−8.9 ± 35.6	[−26.1 to 8.2]	N/A
BMI, kg/cm^2^	30.7 ± 4.8^d^	29.7 ± 4.6^b^	29.5 ± 4.6^c^	29.6 ± 4.3^b^	−1.0 ± 0.8	[−1.4 to −0.7]	N/A
Weight, kg	86.6 ± 15.0^d^	83.7 ± 13.9^b^	82.7 ± 13.8^c^	83.6 ± 13.7^b^	−3.0 ± 2.3	[−4.1 to −1.8]	N/A

*KOOS* Knee injury and Osteoarthritis Outcome Score, *ADL* Activities of daily living, *QoL* Quality of life, *EQ 5D EuroQoL-5D*, *BMI* Body mass index, *MDC* Minimal detectable change, *VAS* Visual analogue scale, *CI* Confidence interval

^a^Change indicates absolute change from baseline to week 9. All data presented as mean ± standard deviation for participants completing the intervention and had baseline data. Minimal detectable change values drawn from previous estimations [43]

^b^*n* = 1 missing data relative to number of participants in each timepoint column

^c^*n* = 2 missing data relative to number of participants in each timepoint column

^d^*n* = 3 missing data relative to number of participants in each timepoint column

## Discussion

The results of this study suggest that a full-scale RCT designed to evaluate the effects of a telehealth delivered anti-inflammatory dietary intervention is feasible. Fifty-eight percent of eligible participants enrolled, attendance at scheduled appointments and adherence to the intervention was excellent, and most (86%) participants were satisfied with the intervention expressing a desire to continue the diet beyond the study period. Additionally, worthwhile treatment effects were observed in participants completing the intervention for knee-related symptoms, function and QoL. The drop-out rate of 21% requires attention in the design of a full-scale RCT.

The significant interest to participate in this study by people with knee OA as evidenced by the high recruitment rates, together with the promising changes in important patient-reported outcomes, supports progression to a full-scale RCT. The rate of enrolment (58%) was much higher than previously reported rates for other pilot RCTs recommending progression to a full-scale RCT (35%) and was similar to other trials of dietary interventions [44]. Our recruitment rate of 14 per month was primarily drawn from individuals responding to a study advert in a newsletter sent out to all patients with knee and hip OA in an existing registry (n ~ 2000) [45]. This rate suggests that a full-scale RCT of approximately 150–200 participants (estimated sample size to achieve 80% power based on a between-group difference of 10 points on the KOOS with a standard deviation of 15) [39, 46] could be recruited over a 12–18 month period [39, 46]. Avenues to increase the recruitment rate could include engaging with hospital, orthopaedic and physiotherapy clinics to directly notify existing patients of the study. This may be required with the addition of a control arm, which may mitigate the desire to participate. Alternative trial designs such as within-subject cross-over design could overcome this potential barrier to recruitment.

Telehealth delivery of the dietary intervention was supported by this feasibility study with excellent attendance at telehealth appointments (99%) and remote completion of 3-day food diaries and other patient-reported outcomes (98%). E-mail and text-message reminders, together with accessibility and flexibility of scheduling, helped to facilitate the high attendance and completion rates, with telehealth delivery considered acceptable and similarly effective to in-person delivery in the qualitative interview feedback. Eighty-six percent of participants interviewed stated they were satisfied with the dietary intervention and would continue following the diet beyond the study period. Two (9%) participants reported adverse events – one related to constipation due to changed eating habits and the other an injury sustained unrelated to the diet. The risk of constipation was discussed in the initial education session as it is a known adverse outcome for diets higher in protein and fat, however, more overt preventive strategies may need to be implemented in future trials. One such strategy to implement would be encouraging the intake of dietary fibres, which were not increased from baseline during the feasibility study. This has the dual benefit of potentially decreasing the adverse side effects of constipation and reducing the development of moderate and severe knee pain, which has been associated with low fibre intake [47, 48].

Participants reported being generally adherent to the anti-inflammatory diet, 27% were adherent everyday and 68% were adherent most of the time. This self-reported adherence data was supported by evidence of macronutrient consumption changes. Carbohydrate and refined grain intake significantly decreased, while dietary fats, mostly in the form of MUFAs and PUFAs, significantly increased. While average protein intake decreased by approximately eight grams, this was not statistically significant, and remained above (>1.0 g/kg/day) the recommended protein intake in older adults of 0.8 g/kg/day [48]. Participant feedback during qualitative interviews highlighted that the range of foods available ensured adequate satiety, with the provided example meal plans and recording of dietary intake aiding adherence. Furthermore, due to the experimental nature of the anti-inflammatory diet, more frequent and rigid nutrition counselling could be provided to future participants to ensure maintenance of restricted carbohydrate intake, particularly during longer intervention periods, with evidence that more carbohydrates were consumed the longer participants were enrolled (Fig. 2).

Participant retention is one aspect that requires attention in the design of a full-scale RCT. Strategies to improve a drop-out rate of 21% are needed, particularly as retention to longer-follow-ups may be more problematic. Although other trials have reported a similar drop-out rate (20%) for a dietary intervention, this was over a 6-month follow-up period [8]. Of the participants who withdrew, three were directly related to the study protocol and three were due to other personal matters unrelated to the diet. It is worth noting that the study was conducted in the middle of significant government-mandated COVID-19 restrictions in Australia, which may have contributed to a higher than usual drop-out rate. An initial face-to-face appointment prior to randomisation may help to educate prospective participants and allow sufficient time to detail the study requirements and the importance of continued follow-up to minimise drop-out. This may coincide well with collection of objective data (e.g., inflammatory markers, dual-energy x-ray absorptiometry (DXA) body composition, functional performance) in a large-scale RCT, but would also increase travel/time requirements and potentially willingness to enrol.

The promising response in self-reported symptoms, function, QoL and weight loss over a relatively short 9-week intervention period, is consistent with the response to a similar 12-week low-carbohydrate diet (<20 g first 3 weeks, then <40 g thereafter) in knee OA [25]. However, our study differed based on the dietary advice designed by our APD, which aimed to encourage anti-inflammatory foods and discouraged pro-inflammatory foods, rather than having a prescriptive carbohydrate target. Our approach was more feasible for participants and the diet was able to better promote nutrient intake (e.g Omega-3 fatty acids, MUFAs and PUFAs) that have evidence to improve OA symptoms [49]. Importantly, a worthwhile within-group effect (exceeding MDC) was contained within the 95% CI for all KOOS subscales and KOOS_4_ after the 9-week intervention – a longer intervention period or combining the dietary intervention with exercise may enhance the treatment effect [8]. All participants had previously completed an OA specific exercise-therapy program (GLA:D) [45], yet despite this, were still experiencing ongoing pain. In qualitative interviews, participants expressed a desire to complete the diet and exercise interventions simultaneously to maximise outcomes. Previous trials have demonstrated a benefit of combining diet and exercise compared to diet or exercise alone on weight loss in overweight adults with knee OA, but the diet was not anti-inflammatory in nature [8]. In the current study, participants lost an average of 3 kg over the 9-week intervention period, similar to weight loss on normocaloric anti-inflammatory diets such as the Mediterranean diet [25, 26].

A limitation of the current study was that we did not determine acceptable thresholds of feasibility a priori; instead, we chose to explore these aspects to inform the design of future fully powered RCTs. Despite the promising outcomes reported by participants following the anti-inflammatory diet, it is important that the results are not interpreted as definitively supporting an anti-inflammatory diet for knee OA given the small sample size and lack of control group. We enrolled participants based on a symptomatic definition of knee OA and did not screen for joint structure (e.g., radiographic OA). However, clinical criteria for the diagnosis of knee OA does not rely on the presence of structural joint changes [50]. Further limitations of our study design include the lack of diversity in participants enrolled and the relatively short follow-up period, which limited our ability to determine long-erm sustainability. All participants had previously completed the GLA:D program and were motivated to improve their knee and general health, which may not accurately represent individuals with knee OA from the general population. Additionally, due to the inability to conduct face-to-face data collection during COVID-19 restrictions, our study relied on subjective data. Despite successfully pivoting the study in response to COVID-19 restrictions, without blood samples to assess changes in inflammatory markers, it was not possible to confirm whether the improvement in symptoms was mediated by changes in systematic inflammation. We appreciate that the literature surrounding anti-inflammatory properties of foods and diets is often conflicting, and that further research is required to continue to objectively substantiate the anti-inflammatory nature of certain foods and low-inflammatory diets. We guided our anti-inflammatory intervention based on the existing literature of low-inflammatory or anti-inflammatory diets that have been shown to decrease systemic inflammation and improve health outcomes [21]. Future large-scale studies will be able to investigate drivers of symptomatic improvements in response to an anti-inflammatory diet. Other measures that would normally be assessed objectively (e.g., height, weight) were self-reported and we were unable to assess changes in body composition (waist-height ratio, DXA) and functional performance (e.g., sit to stand, walk tests).

## Conclusion

A full-scale trial to evaluate the effectiveness of an anti-inflammatory dietary intervention in knee OA is feasible. The likely worthwhile treatment effects and overwhelming positive feedback towards the telehealth delivered format highlights the potential for an anti-inflammatory diet intervention delivered by telehealth to effectively reduce pain, improve function and quality of life and result in weight loss. Additional strategies to minimise drop-out rates are required.

## Supplementary Information

**Additional file 1. **Anti-inflammatory diet information.

**Additional file 2. **Post-study interview questions and lines of enquiry.

**Additional file 3. **Major themes from post-study interview.

**Additional file 4. **Nutrient and Food Group intake in participants who completed all follow-ups.

**Additional file 5. **Change in Knee injury and Osteoarthritis Outcome Score (KOOS) subscale scores.

## Data Availability

The datasets for the study are available from the corresponding author upon reasonable request.

## Abbreviations

OA: Osteoarthritis
GLA:D: Good life with osteoarthritis from Denmark
KOOS: Knee injury and Osteoarthritis Outcome Score
ADL: Activities of daily living
QoL: Quality of life
RCT: Randomised control trial
EQ 5 D: EuroQol-5D
CHO: Carbohydrate
TEI: Total energy intake
MUFA: Mono-unsaturated fatty acids
PUFA: Poly-unsaturated fatty acids
MDC: Minimal detectable change
