# Supplementary material for: An anti-inflammatory diet intervention for knee osteoarthritis: a feasibility study
Source: BMC Musculoskelet Disord. 2022 Jan 13;23:47. doi: 10.1186/s12891-022-05003-7 (PMC8757404; doi:10.1186/s12891-022-05003-7)
Supplement: Supplementary file 1 — Additional file 1. Anti-inflammatory diet information. [file 12891_2022_5003_MOESM1_ESM.pdf]

<u>ANTI-INFLAMMATORY DIET INFORMATION</u>

Inflammation occurs when chemicals from white blood cells are released into the blood stream, targeting affected tissues. This reaction occurs to try and protect the body from any foreign substances. The release in chemicals increases blood flow to the area that needs to be protected – in this case, the knee. These chemicals can cause fluid to leak into affected tissue which results in common symptoms of inflammation (redness, swelling and pain) and can contribute to the deterioration of cartilage. It is known that foods can contain properties that either help to improve/reduce inflammation, or can promote inflammation (cause greater swelling and pain).

It is likely that you may have heard of many foods that can be anti-inflammatory. Our anti-inflammatory diet will be made up of many foods that have anti-inflammatory properties as well as many other foods; however, the main premise of this diet is to consume **wholefoods** and **good fats**, and <u>limit consumption</u> of **processed and refined foods.**

Wholefoods which we encourage you to consume are lean meats, fish, eggs, dairy, fruit, vegetables, nuts and seeds. These foods contain lots of nutrients which are not only essential for the body, but may also help to reduce inflammation such as antioxidants, vitamins and minerals. Consuming a wide variety of vegetables and fruits will provide you with these nutrients. **Good fats** are those which have a high content of ***omega 3*** such as olive oil.

Foods which we are encouraging you **limit consumption** of are **processed and refined foods** such as foods which are/contain refined carbohydrates, sugar, processed foods and meats and junk food. Examples of these foods are bread, pasta, cereals, energy bars, spam, bakery treats, soft drinks, confectionary, potato chips/potato chip alternatives, margarine etc.

On the following pages we have included some tools which may help you with adherence to the anti-inflammatory diet. There is an **example meal plan, shopping list and tips**. You are not required to follow the example meal plan directly, or purchase everything that is on the shopping list, they are just ideas to help you.

Please do not hesitate to contact us if you have any questions or queries throughout the study! We are here to help!

EXAMPLE 2-WEEK MEAL CYCLE

Week 1

	Monday	Tuesday	Wednesday	Thursday	Friday	Saturday	Sunday
**Breakfast**	Natural Greek low-fat yoghurt with berries	Scrambled eggs with spinach and avocado	Mixed berry smoothie	Natural Greek low-fat yoghurt with berries	Scrambled eggs with spinach and avocado	Mixed berry smoothie	Natural Greek low-fat yoghurt with berries
**Snack**	Small handful of walnuts	Natural Greek low-fat yoghurt with berries	Pear	Small handful of walnuts	Natural Greek low-fat yoghurt with berries	Pear	Small handful of walnuts
**Lunch**	Roast vegetable salad	Can of tuna with salad (spinach, cucumber, cherry tomatoes and olive oil)	Leftover vegetable soup	Can of tuna with salad (spinach, cucumber, cherry tomatoes and olive oil)	Left over stuffed capsicums	Salmon with cauliflower rice bowl	Chicken with salad
**Snack**	Almond meal blueberry muffin	Small handful of almonds	Small slice of frittata	Almond meal blueberry muffin	Small handful of almonds	Small slice of frittata	Almond meal blueberry muffin
**Dinner**	Chicken with broccoli, mushrooms and asparagus	Vegetable soup	Baked snapper with steamed green vegetables	Stuffed capsicums (made with turkey mince)	Garlic prawns with zoodles (spiralised zucchini)	Chicken saganaki with Greek salad	Slow-cooked beef cheek and eggplant casserole with Greek salad

Recipes provided:

- Roast vegetable salad: page 11
- Almond meal blueberry muffins: page 12
- Mixed berry smoothie: page 13
- Garlic prawns with zoodles: page 14
- Chicken Saganaki: page 15
- Slow-cooked Beef Cheek and Eggplant Casserole: page 16
- Greek salad: page 17

Week 2

	Monday	Tuesday	Wednesday	Thursday	Friday	Saturday	Sunday
**Breakfast**	Scrambled eggs with spinach	Mixed berry smoothie	Natural Greek low-fat yoghurt with berries	Scrambled eggs with spinach	Mixed berry smoothie	Natural Greek low-fat yoghurt with berries	Scrambled eggs with spinach
**Snack**	Small handful of almonds	Small slice of frittata	Almond meal blueberry muffin	Small handful of almonds	Small slice of frittata	Almond meal blueberry muffin	Small handful of almonds
**Lunch**	Can of tuna with salad (spinach, cucumber, cherry tomatoes and olive oil)	Leftover vegetable soup	Can of tuna with salad (spinach, cucumber, cherry tomatoes and olive oil)	Left over stuffed capsicums	Salmon with cauliflower rice bowl	Chicken with salad	Roast vegetable salad
**Snack**	Natural Greek low-fat yoghurt with berries	Pear	Small handful of walnuts	Natural Greek low-fat yoghurt with berries	Pear	Small handful of walnuts	Natural Greek low-fat yoghurt with berries
**Dinner**	Vegetable soup	Baked snapper with steamed green vegetables	Stuffed capsicums (made with turkey mince)	Garlic prawns with zoodles (spiralised zucchini)	Chicken saganaki with Greek salad	Slow-cooked beef cheek and eggplant casserole with Greek salad	Chicken with broccoli, mushrooms and asparagus

Extra advice to assist you in following the meal plan:

Main meals – if you do not like a component of a main meal (e.g., fish or seafood), you may **substitute** it for **another source of protein**. Consult your Dietitian about this if you have any questions regarding options.

Sides – If you wish to replace a side for something else, **substitute it for the same category of food** (vegetables for vegetables, do not swap vegetables for carbohydrates), e.g. if you don't like broccoli you may have another source of leafy greens or vegetables, however do not replace with rice.

Yoghurt – Where Greek Yoghurt is listed, you may have it plain or with nuts or some fresh fruit. Add cinnamon for flavour.

Nuts – All nuts should be raw and unsalted. We highly recommend almonds and walnuts.

SHOPPING LIST

Meat
Beef
Lamb
Pork
Chicken
Liver
Brains
Kidneys
Heart

Drinks
Water
Coffee
Tea
Bone broth
Soda water

Nuts
Almonds
Walnuts
Brazil Nuts
Hazelnuts
Macadamias
Pecans

Vegetables
Cabbage
Cauliflower
Broccoli
Brussel Sprout
Asparagus
Zucchini
Eggplant
Olives
Spinach
Silverbeet
Mushrooms
Cucumber
Lettuce
Onions
Capsicums
Tomatoes

Dairy
Full fat milk
Cream
Cheese
Cottage Cheese
Greek Yoghurt

Non-dairy alternatives
Coconut milk/cream
Nut butters

Seeds
Flaxseed
Chia
Pumpkin
Sunflower
Sesame

Flour
Almond Flour
Coconut Flour

Fats & Sauces
Olive Oil
Avocado Oil
Coconut Oil
Mayonnaise
Mustard
Pesto

Sweet
Stevia
Xylitol
Erythritol

Fish/Seafood
Cod
Crab
Lobster
Halibut
Mackerel
Mussels
Oysters
Plaice
Salmon
Sardines
Scallops
Shrimp
Trout
Tuna

Other
Avocado
Eggs
Spices
Herbs
Lemon and Lime juice

Fermented Foods
Kimchi
Kombucha
True Pickles
Sauerkraut

Fruits
Apples
Apricots
Peaches
Pears
Oranges
Berries
