# Supplementary material for: An anti-inflammatory diet intervention for knee osteoarthritis: a feasibility study
Source: BMC Musculoskelet Disord. 2022 Jan 13;23:47. doi: 10.1186/s12891-022-05003-7 (PMC8757404; doi:10.1186/s12891-022-05003-7)
Supplement: Supplementary file 2 — Additional file 2. Post-study interview questions and lines of enquiry. [file 12891_2022_5003_MOESM2_ESM.docx]

**Additional file 2.** All post-study qualitative questions and enquiry logic

| **Question** | **Enquiry Logic** |
| --- | --- |
| How are your knee symptoms after the completion of the study? Do you feel it changed over the study period? What factors do you believe influenced the change, if any? | Identifies changes in knee symptoms over the intervention period and explores what factors the participants feel impacted the knee pain. |
| What was your overall experience with the study? What did you like? What did you dislike? | Identifies how participants generally felt about participating in the study, and if there are any specific aspects which they did or did not like. |
| How did you find the recruitment process? Being provided with study documentation and information? regarding scheduling appointments? Phone v e-mail contact? | Identifies any issues that may have arisen during the recruitment process and receiving the required documentation. |
| Do you think telemedicine based dietary appointments were lesser, equal or superior to face to face consultations? Why? | Identifies if participants think that telemedicine is an effective way to conduct dietary consultations compared to traditional face-to-face consultations. |
| How did you find the time commitment for the following: dietary appointments? for the questionnaires? For the diet itself? | Establishes if participants felt any part of the study had too big of a time commitment to complete it. |
| How did you find the written dietary information? How could it be improved? | Identifies if participants felt like they received enough written supplementary information on the diet and any changes that could be made to those resources for future studies. |
| How did you find the zoom and follow-up dietary phone calls? What aspects did you like? What aspects did you not like? | Establishes whether participants liked using Zoom or phone calls to have the dietary appointments and if there were any specific reasons as to why or why not. |
| At each appointment we asked you to rate your adherence to the diet intervention. Now considering the entire 9 weeks – how do you rate your overall adherence? Did you have any difficulty following the dietary intervention? Why/ Why not ? Were there factors that contributed to you following/ not following the diet? What were some things that were helpful, or made it easier, and what were some of the things that made it more challenging? | Identifies how participants perceived their overall adherence to the anti-inflammatory diet across the study period. Also identifies any factors that may have influenced the adherence of individuals to the diet. |
| How did you find the questionnaires, and the specific questions asked? Were they relevant for you? How could they be improved? How did you find completing them over e-mail? | To determine if the validated questionnaires were relevant to the participants, and how they need to be changed to suit this sample population better for future studies. |
| Did you have any difficulty filling in the 3-day food diary? How could monitoring and adherence of your diet be improved? | To determine if there were any barriers or difficulties when recording 3-day food diaries. |
| Did you enjoy the diet? Why or why not? What aspects of the diet did you enjoy most? | Establishes whether participants enjoyed following the anti-inflammatory diet and the specific aspects of the diet which participants did enjoy. |
| Was the expense of following the diet more or less than your typical shopping expense? What did you find made it more or less expensive? | Identifies if participants were spending more money when following the anti-inflammatory diet compared to normal which may lead to a barrier of adherence. |
| What did you find was challenging to change about your diet? Were there any particularly difficult parts that you found hard to include in or restrict from your diet? Why? | Identifies challenges that participants experienced when changing their diet, including any specific foods or food groups and why they these were difficult to incorporate or not. |
| Did you feel satisfied while following the diet? (satiety, were you ever hungry or felt deprived?) | Identifies if participants felt satisfied when following the anti-inflammatory diet. |
| Are you likely to continue the diet? Why/Why not? | Establishes whether participants intended on following the anti-inflammatory diet after the study period and provides insight as to why they may or may not continue, as well as any changes they may make to the diet. |
| What other information would have you liked to have received? | Identifies any additional materials or resources that participants would have liked to receive before the commencement of the study. |
| Do you think COVID-19 impacted upon how you adhered to the diet? In what ways did it impact on your adherence? | Identifies if participants felt that COVID-19 impacted on their adherence to the anti- inflammatory diet and why their adherence may have changed due to COVID-19. |
| Compared to doing exercises for your knee OA, was the diet more or less effective at helping your pain/ function? Was it harder/easier to follow diet compared to exercise program/GLAD? | Determines which program participants believe is more effective at improving knee pain and function and which program is easier to follow. |
| Overall, what have you learnt as a result of your participation in this study? How could it be improved? | Establishes what participants have taken from participating in the study, and any future recommendations they have to further improve future studies. |
| Any other general feedback? | Identifies any other general feedback participants had about the study as a whole, or specific aspects that were not addressed throughout the rest of the interview. |
