# Supplementary material for: An anti-inflammatory diet intervention for knee osteoarthritis: a feasibility study
Source: BMC Musculoskelet Disord. 2022 Jan 13;23:47. doi: 10.1186/s12891-022-05003-7 (PMC8757404; doi:10.1186/s12891-022-05003-7)
Supplement: Supplementary file 3 — Additional file 3. Major themes from post-study interview. [file 12891_2022_5003_MOESM3_ESM.docx]

**Additional file 3.** Major themes from post-study interview

| Theme | Sub-theme | Supporting quotes |
| --- | --- | --- |
| Accessibility of telemedicine dietary consultations. | Travel: Not having to travel to attend the consultations saved time.  Flexibility: The flexibility of using telemedicine to deliver consultations. | *“That would be an hour and a half drive for me.”*  *“I suppose the fact that you didn’t have to travel to an appointment and find extra time to put in was good.”*  *“You don’t have to drive somewhere, particularly if you’re working. It’s way more time efficient.”*  *“It was good to have some flexibility if a time didn’t suit.”*  *“Yep. That was good, because I’m a shift worker, so it was good to have some flexibility.”*  *“I can choose to be seen or not seen, I can choose to be heard or not heard.”* |
| The use of telemedicine compared to face-to-face consultations for dietary appointments. | Effectiveness of telemedicine: Views generally expressed that telemedicine is equally as effect as face-to-face consultations.  Improvements for dietary consultations delivered via telemedicine. A common suggestion was to conduct the initial consultation face-to-face, with follow-up conducted via telemedicine. | *“Probably equal, in this case.”*  *“I would say equal. I didn’t have any issues.”*  *“I don’t have any problems with that at all. … Well, I don’t see that there’s really very much difference.”*  *“Probably, initially, there needs to be a face-to-face consultation.”*  *“If possible, in the future, you have the one-off interview, face-to-face, do all that and the rest of it would just be zoom.”*  *“It was a good substitute for face-to-face, but if you’re going to be developing a rapport with a new dietician or changing doctors, I think it’s better to see them first up.”*  *“…in an ideal world, there would be a combination sort of pre- and post-* [face-to-face] *but you could do the check-ins via telehealth.”* |
| Challenges and experiences of including/excluding certain foods. | Including foods: Challenges came from increasing consumption of certain foods such as olive oil. Participants also enjoyed having recipes to include these foods.  Excluding foods: Challenges with excluding foods arose from not wanting to, or from usually including these foods in their typical diet. | *“I found getting the amount of seeds in everyday challenging and also, um, up to the four tablespoons of olive oil a day. I really struggled.”*  *“I had to cut them* [carbohydrates] *out… I resented cutting them out.”*  *“I do eat a lot of legumes normally, so I did really miss not having, you know, beans with my lunch and stuff like that.”*  *“I very, very much miss though having the cereal and the porridge and pasta.”*  *“Probably just rethinking lunchtime because normally at lunch we’d just have a sandwich”* |
| Overall adherence to anti-inflammatory diet and influencing factors. | Overall adherence to the anti-inflammatory diet: Overall adherence was considered high across the study period.  Factors influencing adherence: Being in lockdown, noticing improvement in knee pain/symptoms, not feeling hungry between meals and enjoying the variety helped improve adherence. | *“I was probably about 95% adherent… maybe 98%.”*  *“In percentage-wise, probably 80.”*  *“Perfect… I was perfect. I didn’t do anything naughty at all.”*  *“I think every day I adhered to it.”*  *“I was really pleased we were in lockdown, I must admit. I think that would have been much more difficult to maintain going in and out of work every day.”*  *“I suppose, when I was having positive results out of it, when my knee was not hurting as much, when I could actually see that the swelling on my knee.. were going down, plus seeing he weight loss and everything. So, it wasn’t hard to adhere to it.”*  *“I think the fact that after he first ten days, I felt so much better.”*  *“yes, I did. There was a good variety of different foods and I think just trying to try new things, so finding new recipes. It just makes it a bit more interesting”*  *“Actually, throughout the diet I found that, when I adhered to it, I was far less likely to eat between meals, which was good.”*  *“Well, I enjoyed following a different way of eating”*  *“I like the menu. I enjoyed the menu”* |
| Ongoing adherence to the anti-inflammatory diet | Continuing the diet post-study period: Continuation of the anti-inflammatory diet after the end of the study was popular, particularly if there was an improvement in knee symptoms or overall health. | *“Yes. I’m going to continue the diet because I felt better.”*  *“… I think I’ll probably, mostly try and continue with it and see if I get more improvement.”*  *“Yeah, I will. Because, clearly my pain levels have diminished and, yeah, I’m not a big carbohydrate eater anyway, so if I don’t have those foods it won’t bother me.”*  *“I will continue with it because I think that it has made a difference.”* |
| Differences in following the anti-inflammatory diet to completing exercise based intervention | Easier to follow: The anti-inflammatory diet was generally considered to be easier to follow than the GLA:D program.  Participating in both interventions concurrently: Participants generally stated completing both interventions at the same time would have been beneficial. | *“.. The diet was easier to follow. The diet was much easier to follow.”*  *“It was easier to follow the eating plan. Because I did GLAD post a total knee replacement. So, that was hard for pain.”*  *“…, It* [diet] *was much easier because I find the exercises very tiring and hard.”*  *“I think they’re both easy to adhere to because the exercises obviously are going to help strengthen and whatever and the anti-inflammatory diet is going to help maybe lubricate”*  *“Yeah, absolutely.”*  *“I often have had that feeling and putting something like the GLAD program and diet together, it’s a step in the right direction in that we should be treated as a whole person.”*  *“That would be a really interesting thing to do, actually, to see… because if the pain’s genuinely backing off from the diet, then you might actually get much better results with what you were trying to do with the exercises.”* |
| Overall treatment satisfaction after following the anti-inflammatory diet. | Treatment satisfaction: Common feedback was participants noticed less swelling and inflammation and improved mobility. | *“…I could tell after day three that it was different. Like, reduced pain, particularly in the night-time.”*  *“I’d have to say that within ten days of starting the eating program, fifty percent of the discomfort went.”*  *Yes. I’m able to walk a lot further now… I mean, I don’t really feel that I need to have a knee reconstruction now.”*  *“There is less swelling around my knee, clearly since I’ve been on this program.”*  *“to look at my knees, the inflammation has subsided. Physically, I can actually see that.”* |
