# Supplementary material for: An anti-inflammatory diet intervention for knee osteoarthritis: a feasibility study
Source: BMC Musculoskelet Disord. 2022 Jan 13;23:47. doi: 10.1186/s12891-022-05003-7 (PMC8757404; doi:10.1186/s12891-022-05003-7)
Supplement: Supplementary file 4 — Additional file 4. Nutrient and Food Group intake in participants who completed all follow-ups. [file 12891_2022_5003_MOESM4_ESM.docx]

**Additional file 4.** Nutrient and Food Group intake in participants who completed all follow-ups (n=22)

|  | **Baseline** | **Week 3** | **Week 6** | **Week 9** | **Change†** | **95 % CI** | **p-value** |
| --- | --- | --- | --- | --- | --- | --- | --- |
| **Total Energy (kcal)** | 1858.8 ± 645.6 | 1746.4 ± 475.7 | 1790.6 ± 458.1 | 1789.7 ± 567.3 | -69.0 | [-308.8 to 170.7] | .564 |
| **Total Energy (kJ)** | 7777.1 ± 2701.3 | 7306.8 ± 1990.4 | 7491.8 ± 1916.6 | 7488.2 ± 2373.5 | -288.8 | [-1291.9 to 714.1] | .564 |
| **CHO (g)** | 202.9 ± 98.4 | 100.8 ± 46.3 | 126.6 ± 55.2 | 138.1 ± 71.9 | -64.8 | [-104.9 to -24.7] | .004 |
| **CHO (% TEI)** | 42.6 ± 10.1 | 23.1 ± 8.4 | 27.7 ± 9.9 | 29.3 ± 8.9 | -13.3 | [-18.2 to -8.4] | .000 |
| **Protein (g)** | 94.8 ± 39.4 | 100.8 ± 47.1 | 97.5 ± 47.0 | 86.7 ± 24.7 | -8.1 | [-20.9 to 4.8] | .233 |
| **Protein (% TEI)** | 20.7 ± 5.7 | 23.2 ± 5.7 | 21.8 ± 5.6 | 20.6 ± 5.0 | -0.1 | [-2.8 to 2.6] | .947 |
| **Total Fat (g)** | 66.1 ± 24.6 | 93.4 ± 27.2 | 92.0 ± 26.5 | 88.5 ± 32.6 | 22.5 | [7.7 to 37.3] | .007 |
| **Total Fat (% TEI)** | 31.6 ± 8.1 | 47.7 ± 8.8 | 45.7 ± 9.1 | 43.8 ± 8.7 | 12.2 | [7.1 to 17.4] | .000 |
| **SFA (g)** | 26.3 ± 11.4 | 30.3 ± 10.4 | 32.0 ± 11.1 | 30.0 ± 10.8 | 3.8 | [-1.9 to 9.4] | .203 |
| **SFA (fat ratio %)** | 43.4 ± 12.4 | 35.3 ± 7.1 | 38.4 ± 7.2 | 37.7 ± 8.1 | -5.7 | [-11.0 to -0.5] | .044 |
| **SFA (%TEI)** | 12.7 ± 5.5 | 15.6 ± 5.4 | 16.1 ±5.6 | 15.1 ± 5.4 | 2.4 | N/A | N/A |
| **MUFA (g)** | 23.4 ± 10.5 | 39.6 ± 14.5 | 38.7 ± 14.6 | 35.3 ± 14.4 | 12.0 | [5.1 to 18.9] | .003 |
| **MUFA (fat ratio %)** | 38.8 ± 7.2 | 45.7 ± 7.4 | 44.1 ± 7.0 | 43.1 ± 6.0 | 4.3 | [1.3 to 7.4] | .012 |
| **MUFA (%TEI)** | 11.3 ± 5.1 | 20.4 ± 7.5 | 19.5 ± 7.3 | 17.8 ± 7.2 | 6.5 | N/A | N/A |
| **PUFA (g)** | 10.6 ± 6.0 | 16.1 ± 6.1 | 15.2 ± 8.2 | 16.5 ± 11.7 | 6.0 | [1.7 to 10.2] | .019 |
| **PUFA (fat ratio %)** | 17.8 ± 7.6 | 19.0 ± 4.0 | 17.4 ± 6.7 | 19.2 ± 6.8 | 1.4 | [-1.7 to 4.5] | .391 |
| **PUFA (%TEI)** | 5.1 ± 2.9 | 8.3 ± 3.1 | 7.6 ± 4.1 | 8.3 ± 5.9 | 3.2 | N/A | N/A |
| **Vegetables (serve)** | 3.53 ± 2.07 | 5.63 ± 2.35 | 5.68 ± 2.33 | 4.73 ± 1.98 | 1.20 | [-2.2 to 0.30] | .131 |
| **Fruit** | 1.38 ± 1.13 | 1.12 ± 0.50 | 1.27 ± 0.79 | 1.51 ± 0.89 | 0.13 | [-0.83 to 0.35] | .411 |
| **Fibre (g)** | 25.4 ± 9.7 | 25.4 ± 7.6 | 26.3 ± 6.9 | 25.6 ± 9.0 | 0.2 | {-3.95 to 4.22] | .947 |
| **Grains (serve)** | 5.56 [2.9 to 6.7] | 0.84 {0.10 to 1.6] | 0.88 [0.19 to 3.7] | 1.13 [0.23 to 3.1] | -4.4 | N/A | N/A |
| **Wholegrain (serve)** | 1.25 [0.0 to 2.6] | 0.11 [0.0 to 1.1] | 0.29 [0.0 to 1.4] | 0.33 [0.0 to 1.2] | -0.92 | N/A | N/A |
| **Refined Grain (serve)** | 3.54 [2.6 to 6.5] | 0.38 [0.0 to 1.4] | 0.88 [0.19 to 2.1] | 0.95 [0.19 to 2.04] | -2.6 | N/A | N/A |

† Change represents absolute change in variable from baseline (week 0) to week 9. All normally distributed data presented as mean ± SD with the 95% confidence interval included. All other data presented as median [Q1 to Q3].

CI, confidence interval; kj; kilojoule, kcal; kilocalorie, CHO, Carbohydrate; TEI, Total Energy Intake; SFA, Saturated Fatty Acid; MUFA, Mono-Unsaturated Fatty Acid; PUFA, Poly-Unsaturated Fatty Acid.
