# Supplementary material for: An anti-inflammatory diet intervention for knee osteoarthritis: a feasibility study
Source: BMC Musculoskelet Disord. 2022 Jan 13;23:47. doi: 10.1186/s12891-022-05003-7 (PMC8757404; doi:10.1186/s12891-022-05003-7)
Supplement: Supplementary file 5 — Additional file 5. Change in Knee injury and Osteoarthritis Outcome Score (KOOS) subscale scores. [file 12891_2022_5003_MOESM5_ESM.docx]

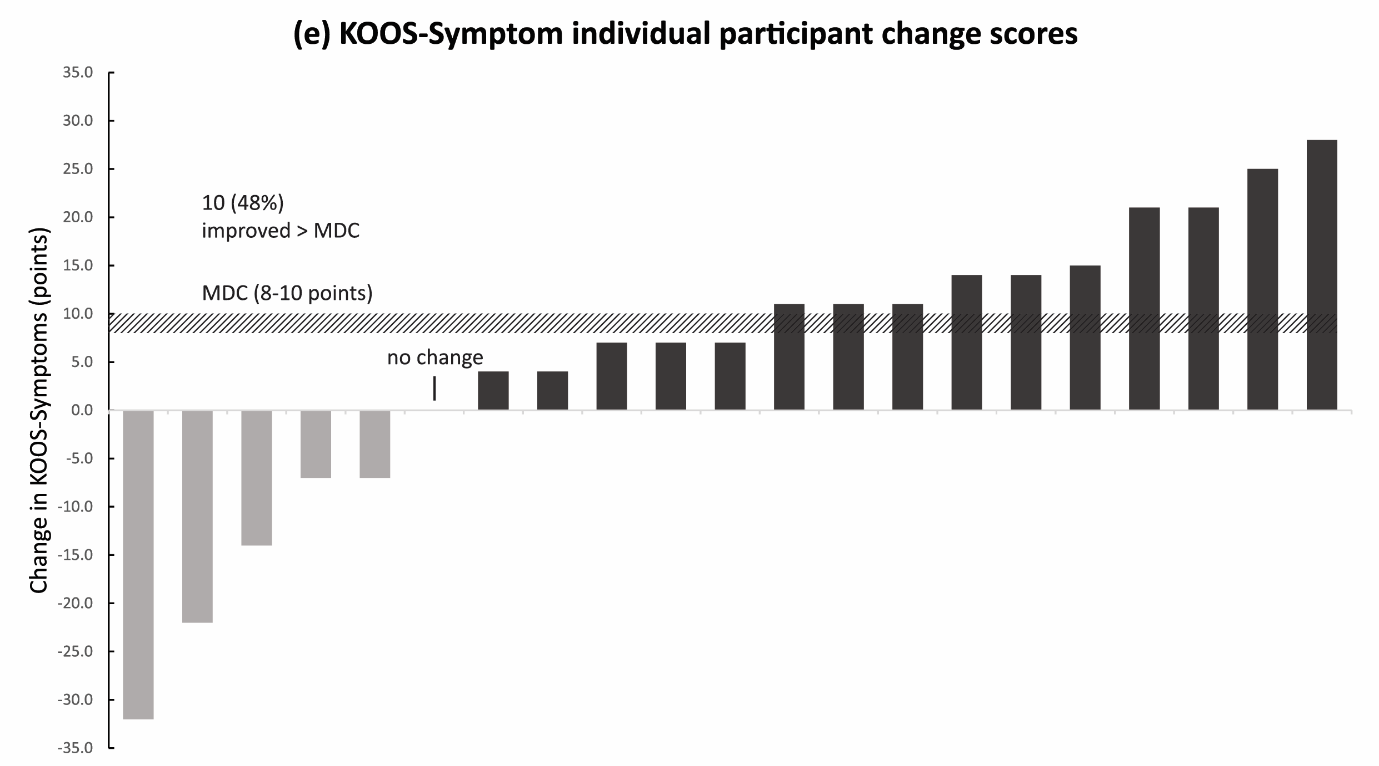

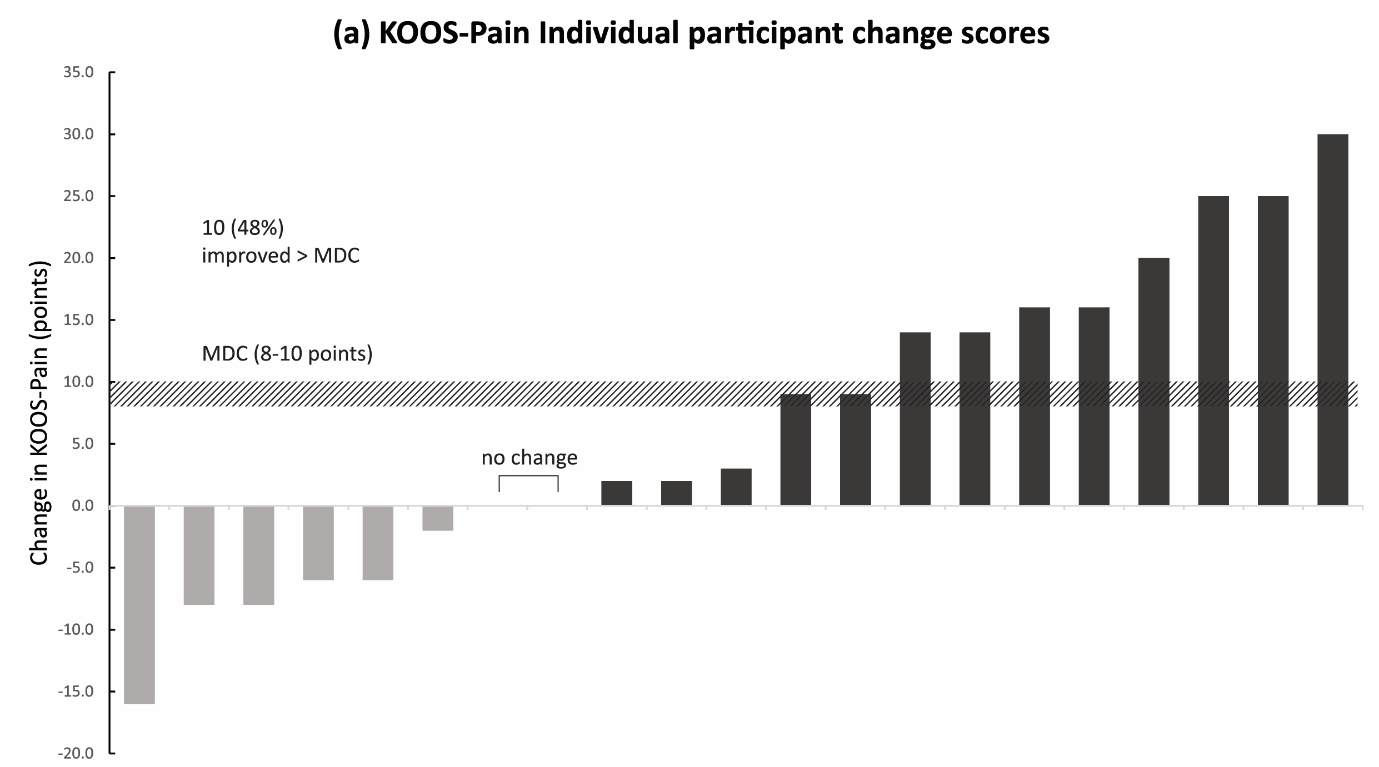
**Additional file 5.** Change in Knee injury and Osteoarthritis Outcome Score (KOOS) subscale scores from baseline to week 9.

**(b) KOOS-Symptoms individual participant change scores**


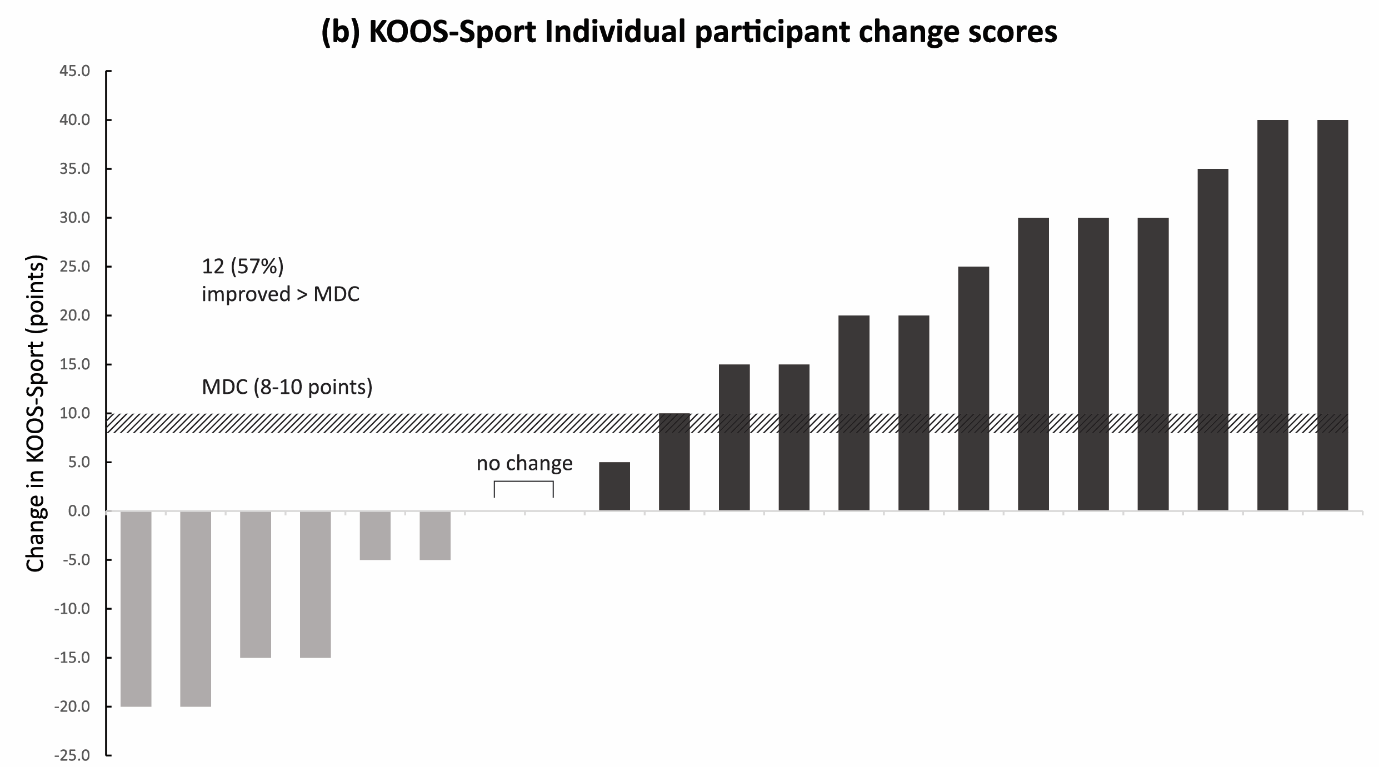

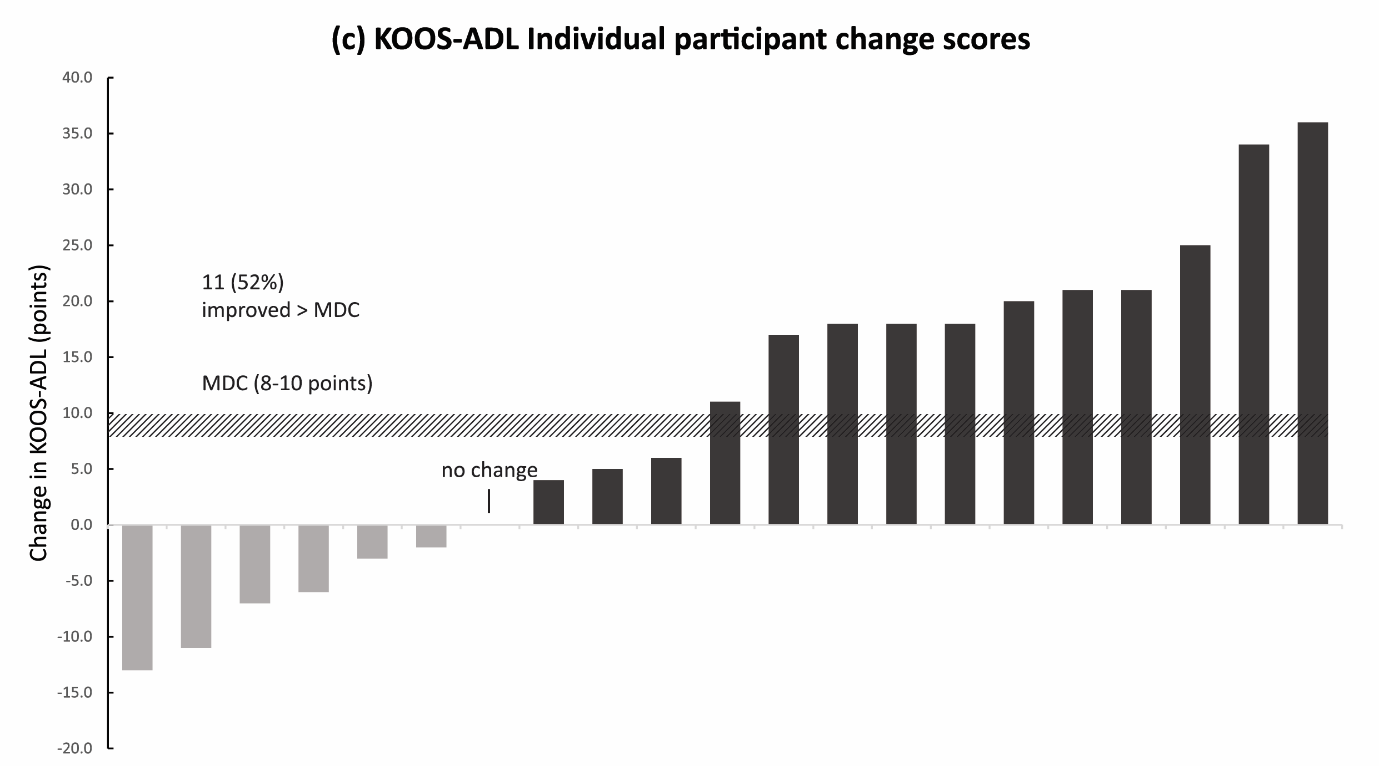


**(d) KOOS-Sport/Recreation individual participant change scores**


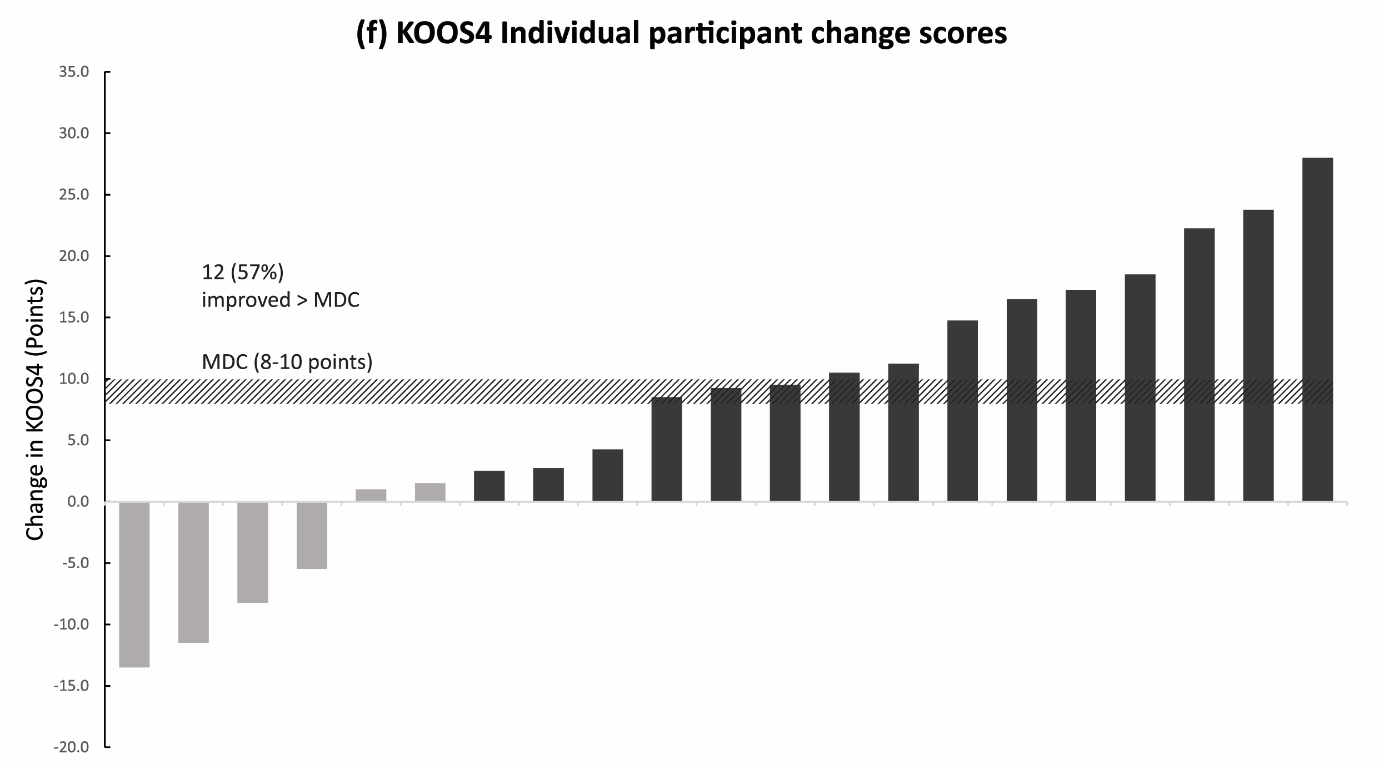

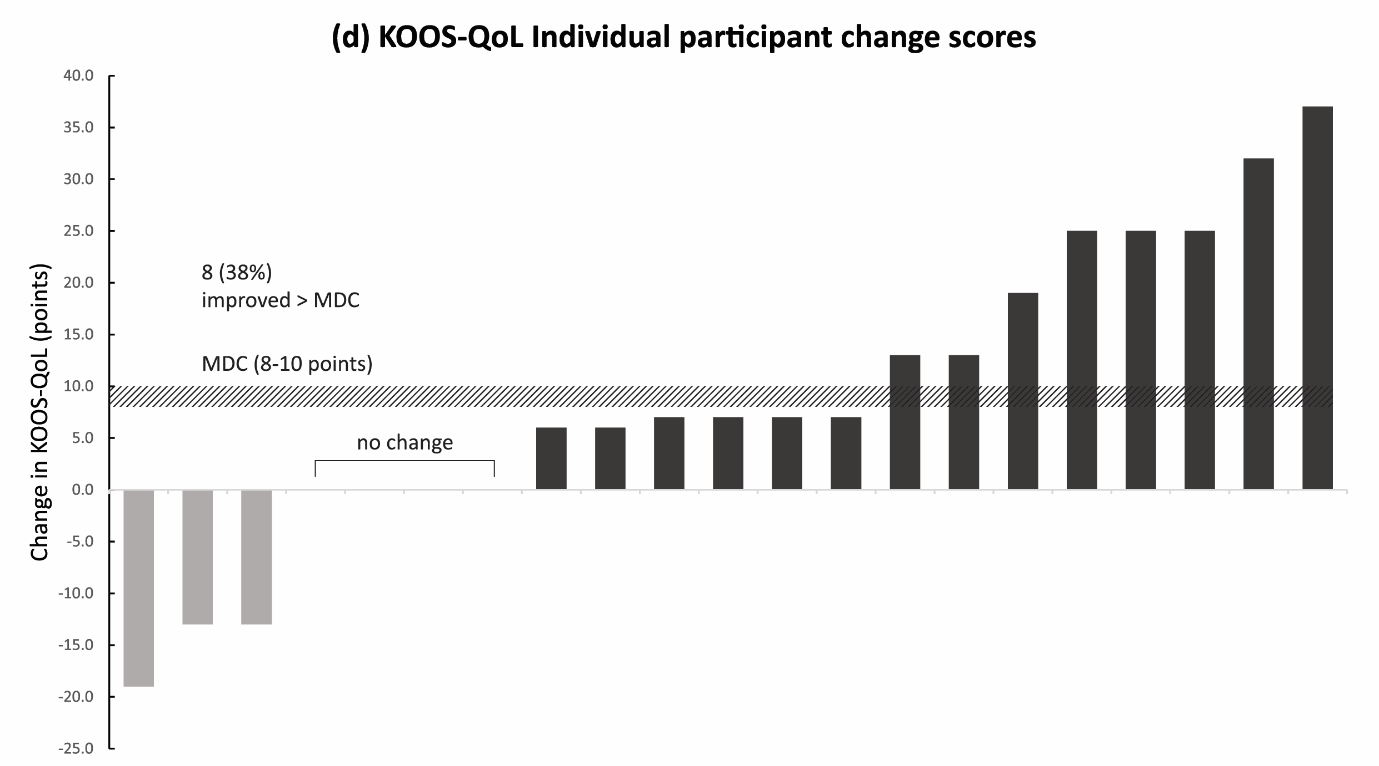


**(e) KOOS-QoL individual participant change scores**
